# A Moderate Low-Carbohydrate Low-Calorie Diet Improves Lipid Profile, Insulin Sensitivity and Adiponectin Expression in Rats

**DOI:** 10.3390/nu7064724

**Published:** 2015-06-11

**Authors:** Jie-Hua Chen, Caiqun Ouyang, Qiang Ding, Jia Song, Wenhong Cao, Limei Mao

**Affiliations:** 1 Department of Nutrition and Food Hygiene, School of Public Health and Tropical Medicine, Southern Medical University, Guangzhou 510515, Guangdong, China; E-Mails: siyanpijiehua@gmail.com (J.C.); oycq108729@163.com (C.O.); songj_lin@163.com (J.S.); 2 Department of Nutrition and Food Hygiene, School of Public Health, Tongji Medical College, Huazhong University of Science and Technology, Wuhan 430030, Hubei, China; E-Mail: dq0306@163.com; 3 Department of Nutrition, The Gillings School of Global Public Health, The University of North Carolina at Chapel Hill, Chapel Hill, NC 27599, USA; E-Mail: caow@unc.edu

**Keywords:** dietary intervention, metabolic syndrome, adiponectin, peroxisome proliferator activator receptor γ, dietary carbohydrate restriction, rats

## Abstract

Calorie restriction (CR) via manipulating dietary carbohydrates has attracted increasing interest in the prevention and treatment of metabolic syndrome. There is little consensus about the extent of carbohydrate restriction to elicit optimal results in controlling metabolic parameters. Our study will identify a better carbohydrate-restricted diet using rat models. Rats were fed with one of the following diets for 12 weeks: Control diet, 80% energy (34% carbohydrate-reduced) and 60% energy (68% carbohydrate-reduced) of the control diet. Changes in metabolic parameters and expressions of adiponectin and peroxisome proliferator activator receptor γ (PPARγ) were identified. Compared to the control diet, 68% carbohydrate-reduced diet led to a decrease in serum triglyceride and increases inlow density lipoprotein-cholesterol (LDL-C), high density lipoprotein-cholesterol (HDL-C) and total cholesterol; a 34% carbohydrate-reduced diet resulted in a decrease in triglycerides and an increase in HDL-cholesterol, no changes however, were shown in LDL-cholesterol and total cholesterol; reductions in HOMA-IR were observed in both CR groups. Gene expressions of adiponectin and PPARγ in adipose tissues were found proportionally elevated with an increased degree of energy restriction. Our study for the first time ever identified that a moderate-carbohydrate restricted diet is not only effective in raising gene expressions of adiponectin and PPARγ which potentially lead to better metabolic conditions but is better at improving lipid profiles than a low-carbohydrate diet in rats.

## 1. Introduction

Metabolic syndrome (MetS) is a collection of medical conditions that can lead to obesity, diabetes, cardiovascular disease and hypertension. The prevalence of MetS is on a rapid rise due to the shifted paradigm of diet and lifestyle and thus has afflicted many people worldwide. It is well known that calorie restriction (CR) improves some markers of MetS [1,2,3], such as blood pressure, blood glucose and plasma cholesterol, as demonstrated in a number of animal models, and humans [3,4].

Besides functioning as primary energy storage reservoirs, adipose tissue also acts as an endocrine organ secreting numerous protein hormones into the circulation [5]. It was reported that a reduction in White Adipose Tissue (WAT) via CR is likely to change the levels of its secreted hormones. Among all the WAT depots, Visceral Adipose Tissue (VAT) is closely related in particular to MetS and thus is a pathogenic fat depot [6]. The VAT compartment secretes adipokines and cytokines that are predisposed to the development of metabolic traits [7]. Adiponectin is an adipokine and mainly expressed in adipose tissues. Adiponectin has been shown as an insulin-sensitive hormone that participates in the regulation of glucose and lipid metabolism. It was found that adiponectin levels rise during CR [8].

Several elements are capable of modulating adiponectin gene expression, most notably peroxisome proliferator activator receptor γ (PPAR-γ) [9], which is expressed in adipose tissues. A response element of PPARγ was discovered on the promoter of adiponectin gene [10]. Treatment with PPARγ agonists induced adiponectin synthesis during adiponeogenesis [11,12,13]. PPARγ can also be upregulated by CR through its antioxidative action [14,15].

The beneficial effect of CR on prevention and treatment of MetS has been widely recognized. The optimal CR diet, however, remains to be identified. Among all the CR diets, CR via manipulating dietary carbohydrate content has attracted increasing interest due to its effectiveness on weight loss, glycemic control, insulin sensitivity and the management of cardiovascular risk factors [16]. However, there is little consensus about the extent of dietary carbohydrate restriction to elicit the optimal result in controlling metabolic parameters. In the present study, 12-week CR by varying dietary carbohydrate content (80% or 60% energy (34% or 68% carbohydrate reduction) of the control diet) was employed in Wistar rats to investigate the changes in metabolic parameters and expressions of adiponectin and PPARγ. The results of the study will provide a new perspective and new scientific evidence for the prevention and treatment of MetS.

## 2. Experimental Section

### 2.1. Animals and Diets

Thirty-six male Wistar rats (two months old, body weight 240–260 g) were obtained from a local supplier for laboratory animals (Laboratory Animal Center of Hubei Province; Wuhan, China). Rats were housed individually under laboratory conditions (12 h day and night lighting cycle, 22 °C ± 2 °C, 50% ± 10% humidity). All experimental protocols were reviewed and approved by Tongji Medical College Council of Animal Care Committee. Rats were randomly assigned into three different groups, *ad libitum* group (AL group; *n* = 12), calorie restriction group 1 (CR1 group; *n* = 12) and calorie restriction group 2 (CR2 group; *n* = 12). All rats had *ad libitum* access to drinking water.

Rat chow diets were prepared based on the Association of Official Analytical Chemists (AOAC) and AIN-93G formulas to meet the nutrient and energy requirements for the growth and development of rats [17]. Rats were fed with these diets for 12 weeks. AL group had *ad libitum* access to the feed whereas CR1 and CR2 group were maintained on a CR regimen with daily access to 80% and 60% of the calorie intake of AL group, respectively (Table 1). Starch was the only nutrient reduced from diets to achieve calorie restriction for CR1 and CR2 groups whereas the amount of other nutrients provided were at the same level as those in the AL group to ensure sufficient nutrients were provided to maintain normal growth of rats. As starch constitutes 58.5% (w/w) of the control diet, 80% and 60% CR correspond to 34% and 68% carbohydrate reduction in CR1 and CR2 group, respectively. The amount of feed consumed by each rat was recorded daily and body weights of rats were monitored weekly. At the end of the 12-week study, rats fasted for 10 h overnight and blood samples were collected. Rats were then sacrificed by decapitation. Serum samples were obtained from all blood samples promptly after blood collection and stored at −80 °C. The peri-epididymal and perirenal adipose tissues were obtained, weighed and frozen immediately in liquid nitrogen prior to storage at −80 °C.

**Table 1:** Composition of rat chow diet *.

Components	AL Group		CR1 Group ^a^		CR2 Group ^b^	
	g/kg Diet	% Energy	g/0.8 kg Diet	% Energy	g/0.6 kg Diet	% Energy
Casein	200.0	20.2	200.0	25.2	200.0	33.8
Vitamin mix **	10.0	-	10.0	-	10.0	-
Mineral mix **	35.0	-	35.0	-	35.0	-
Sucrose	50.0	5.0	50.0	6.3	50.0	8.4
Corn starch	585.0	58.9	385.0	48.6	185.0	31.2
Fiber	50.0	-	50.0	-	50.0	-
Lipid	70.0	15.9	70.0	19.9	70.0	26.6
**Total amount (g)**	1000.0	100.0	800.0	100.0	600.0	100.0
**Total Calorie/kg diet (Kcal)**	3970.0		3170.0		2370.0	
***Energy ratio (%)***						
Protein/Total	20		25		34	
Carbohydrate/Total	64		55		40	
Lipid/Total	16		20		26	

* Abbreviations: AL: *ad libitum*; CR: calorie restriction; ** The composition of vitamin and mineral mix can be referred to AIN-93G; ^a^ CR1 group was provided with 80% of *ad libitum* intake based on AL group; ^b^ CR2 group was provided with 60% of *ad libitum* intake based on AL group.

### 2.2. Biochemical Measurements

Commercial ELISA kit (R & D Systems, Minneapolis, MN, USA) was used to measure serum adiponectin at the baseline and at the end of the study. Fasting blood glucose, fasting serum insulin, serum total cholesterol, fasting triglyceride and serum high density lipoprotein cholesterol (HDL-C) were determined by enzymatic colorimetric analysis using commercialized kits purchased from BioSino Bio-technology and Science Inc., Beijing, China. All the measurements were performed using an automatic enzymatic analyser (BioTek SpectraMax M2, Winooski, VT, USA). Triglyceride level was measured using glycerol-3-phosphate oxidase-p-aminophenazone (GPO-PAP) method. Total cholesterol was determined using cholesterol oxidase-p-aminophenazone (CHOD-PAP) method. After precipitation of very-low-density lipoprotein (VLDL) and low-density lipoprotein cholesterol (LDL-C), HDL-C was measured, as well. Serum LDL-C were then obtained based on Friedewald equation, in which, LDL-C = Total Cholesterol − (Triglyceride/5 + HDL-C) [18]. Blood glucose level was determined by glucose oxidase method. Insulin level was determined with a commercialized radioimmunoassay kit. Homeostasis Model Assessment of Insulin Resistance (HOMA-IR) was calculated based on the relationship between fasting plasma glucose and insulin: HOMA-IR = fasting plasma glucose (mmol/L) × insulin (mIU/L)/22.5 [19].

### 2.3. Semiquantitative Reverse Transcriptase Polymerase Chain Reaction

Total RNA of the epididymal adipose tissue was extracted using Trizol reagent (Promega, Madison, WI, USA). The concentration of RNA was determined using nucleic acid analyser (Eppendorf, Germany). RNA (3 μg) was reverse transcribed in a 25 μL reaction system into complementary DNA which was then amplified using polymerase chain reaction (PCR). The following primers were used for PCR: 5′-TCCCTCCACCCAAGGAAACT-3′ (sense) and 5′-TTGCCAGTGCTGCCGTGATA-3′ (antisense) for adiponectin; 5′-CATCACTATCGGCAATGAGC-3′ (sense) and 5′-GACAGCACTGTGTTGGCATA-3′ (antisense) for β-actin; 5′-TCCGTGATGGAAGACCACTC-3′ (sense) and 5′-CCCTTGCATCCTTCACAAGC-3′ for PPARγ. β-actin served as a loading control.

All PCR reactions contained 0.2 mmol/L dNTP (deoxyribonucleotide triphosphate), 1.5 μL complementary DNA, 0.25 μmol/L of each primer, 1 × PCR buffer, and 0.8 μL Taq polymerase. The following cycling profile was used for PCR reactions: 5 min of denaturation at 95 °C followed by 30 cycles with 1 min at 94 °C, 45 s at 58 °C and 1 min at 72 °C for adiponectin or 35 cycles with 1 min at 94 °C, 40 s at 56 °C and 1 min at 72 °C for PPARγ or 35 cycles with 1 min at 94 °C, 45 s at 59 °C and 1 min at 72 °C for β-actin and the final extension step of 10 min at 72 °C in a PCR Thermocycler (Biometra, Göttingen, Germany).

PCR products were subjected to electrophoresis in 1.5% agarose gels and the results were analyszed by gel imaging and analysis system (Biometra, Göttingen, Germany). The expression levels of adiponectin mRNA and PPARγ mRNA were shown using the absorbance ratio of target mRNA and β-actin mRNA.

### 2.4. Statistical Analysis

All the data were analysed using one-way ANOVA followed by Least Significant Difference (LSD)-test with SPSS version 12 software (SPSS Inc., Chicago, IL, USA). Transformation was applied to correct for unequal variances. Pearson correlation coefficient was used to analyze the linear relationship between adiponectin mRNA level and different parameters, *i.e.*, body weight, blood glucose, total cholesterol, triglyceride, HDL-C and insulin. Similar tests were applied to analyze the linear relationship between PPARγ mRNA level and those parameters. In all tests, a value of *p* ≤ 0.05 was considered statistically significant.

## 3. Results

### 3.1. Calorie Intake and Body Weight

**Figure 1:**
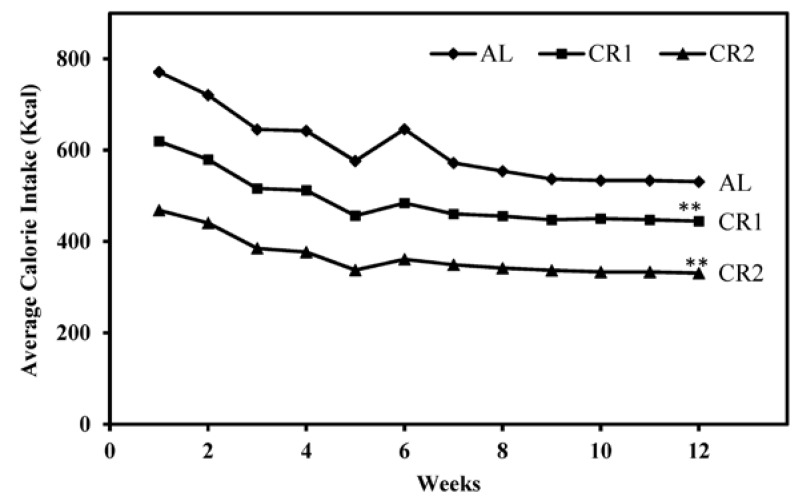
Weekly average calorie intakes of three groups over the 12-week feeding period. Average calorie intake of rats across all groups was shown to be higher at the beginning of the study when rats were fast-growing and then slowly decreased to a plateau with decreasing growth rates of rats. One-way ANOVA followed by LSD-test was used to detect significant differences of the means of the body weights at the end of the study ** *p* < 0.001.

Average calorie intakes of all three groups demonstrated a similar decreasing trend over the 12-week feeding period due to decreased growth rates of rats with maturation (Figure 1). In good agreement with the study design, the ratio of total calorie intake of AL, CR1 and CR2 groups during the feeding period was calculated as 100:81:61.

**Figure 2:**
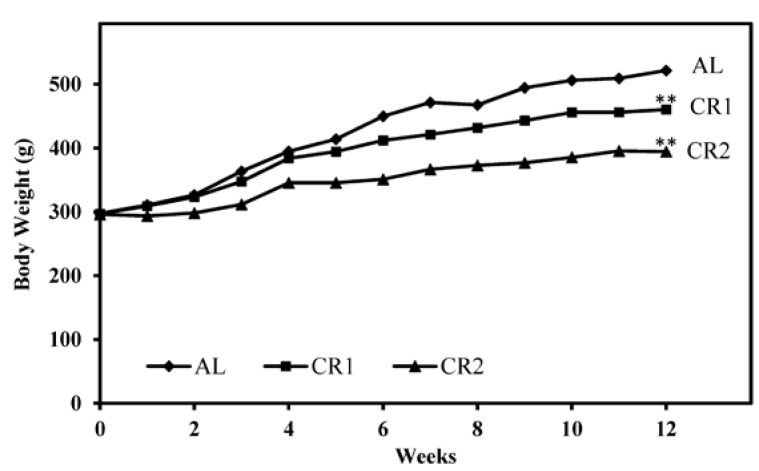
Change of body weights of rats from three groups over the 12-week feeding period. One-way ANOVA followed by LSD-test was used to detect significant differences of the means of the body weights at the end of the study ** *p* < 0.001.

The effect of CR on body weights of rats was found to be significant (Figure 2). Compared to the control group AL, growth rates of rats in CR1 and CR2 group were much slower. Our results indicated that significant differences of body weights of rats among groups were detected in Week 4 (*p* < 0.05) and the differences were greater towards the end of the study (*p* < 0.001).

### 3.2. Changes in Metabolic Parameters

Both 80% (CR1) and 60% (CR2) calorie-restricted diets led to a significant decrease in triglyceride as compared to control diet (AL) (*p* < 0.05; Table 2). However, total cholesterol was found increased in CR2 group (*p* < 0.05) whereas no change was observed in CR1 when compared to AL group (*p* > 0.05; Table 2). Though HDL-C in CR1 and CR2 groups were higher than those in AL group (*p* < 0.05), no significant differences were observed in the ratios of total cholesterol to HDL-C among all the groups (*p* > 0.05). The effect of CR on LDL-C resembled that on total cholesterol which LDL-C increased only in CR2 group (*p* < 0.05).

**Table 2:** Fasting blood lipid, metabolic and insulin responses in rats of different levels of calorie restriction ^†,^*.

Group			mmol/L					
	Triglyceride	Total Cholesterol	HDL-C	LDL-C	Total Cholesterol/HDL-C	Glucose	Insulin (IU/mL)	HOMA-IR ^#^
AL	1.55 ± 0.59	1.47 ± 0.40	0.69 ± 0.16	0.47±0.39	2.14 ± 0.53	5.54 ± 0.98	23.92 ± 8.76	5.59 ± 0.41
CR1	1.19 ± 0.32 ^a^	1.58 ± 0.39	0.82 ± 0.19 ^a^	0.52±0.47	1.95 ± 0.39	5.46 ± 0.67	16.02 ± 9.43	3.88 ± 0.63 ^a^
CR2	0.92 ± 0.13 ^a^	2.09 ± 0.71 ^a^	0.92 ± 0.22 ^a^	0.99±0.61 ^a^	2.25 ± 0.36	5.97 ± 0.98	10.53 ± 7.59 ^a^	2.79 ± 0.58 ^a,b^

^†^ Abbreviations: AL: *ad libitum*; CR: calorie restriction; HDL-C: high density lipoprotein cholesterol; LDL-C: low density lipoprotein cholesterol; * Blood samples were collected at the end of the study; Data was presented as arithmetic mean ± 1 SD (*n* = 12 for each group); ^#^ HOMA-IR (Homeostasis Model Assessment of Insulin Resistance) = Fasting Blood Glucose (mmol/L) × Fasting Insulin (mIU/L)/22.5 [19]; ^a^ *p* < 0.05 *versus* AL group; ^b^ *p* < 0.05 *versus* CR1 group.

No significant difference was detected in blood glucose among all groups (Table 2). Insulin levels decreased by CR which was significant in CR2 compared to AL (*p* < 0.05). Insulin resistance, as presented as HOMA-IR, significantly decreased with increased levels of CR (*p* < 0.05).

**Figure 3:**
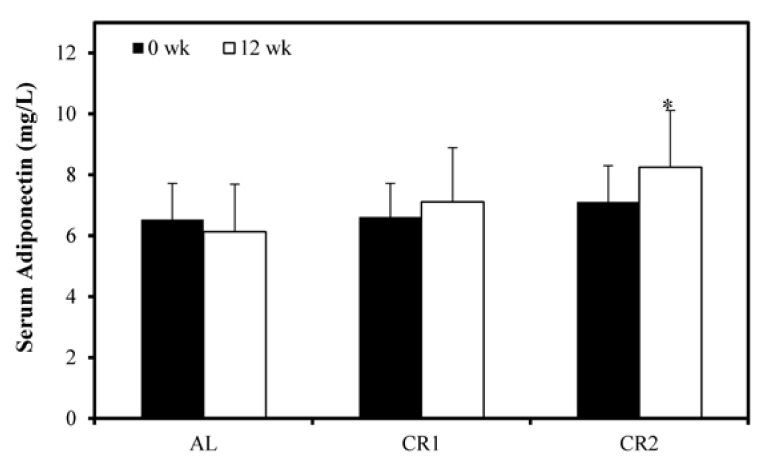
The effect of calorie restriction on serum adiponectin. Serum adiponectin showed no significant difference among all three groups at the beginning of the study. During the 12-week period, only CR2 group demonstrated a significant increase in serum adiponectin level as compared to the baseline. One-way ANOVA followed by LSD-test was used to detect significant differences of the means, * *p* < 0.05.

All groups demonstrated a similar level of serum adiponectin at the beginning of the study (Figure 3). At the end of the study, serum adiponectin was only significantly increased in CR2 group but not in AL and CR1 groups.

CR also led to a decrease in mass of visceral adipose tissue (Table 3). At the end of the 12-week study, wet weights of VAT and its percentage to the total body weight in CR groups were significantly lower than those in AL group (*p* < 0.05). As compared to CR1 group, further calorie restriction in CR2 group resulted in a significantly lower visceral fat mass (*p* < 0.05).

**Table 3:** Effect of calorie restriction on visceral adipose tissue ^†^.

Group	Visceral Fat Mass [g] ^#,^*	Visceral Fat Mass [%] *^,Δ^
AL	16.47 ± 3.76	3.29 ± 0.48
CR1	12.08 ± 3.71 ^a^	2.51 ± 0.68 ^a^
CR2	6.72 ± 2.61 ^a,b^	1.79 ± 0.69 ^a,b^

^†^ Abbreviations: AL: *ad libitum*; CR: calorie restriction; * Data was presented as arithmetic mean ± 1 S.D. *n* = 12 for each group; ^#^ Visceral Fat Mass [g] = total perirenal adipose tissue [g] + total peri-epididymal adipose tissue [g] [20]; ^Δ^ Visceral fat mass [%] = Visceral fat mass/Body weight × 100; ^a^ *p* < 0.05 *versus* AL group; ^b^ *p* < 0.05 *versus* CR1 group.

### 3.3. Gene Expression

**Figure 4:**
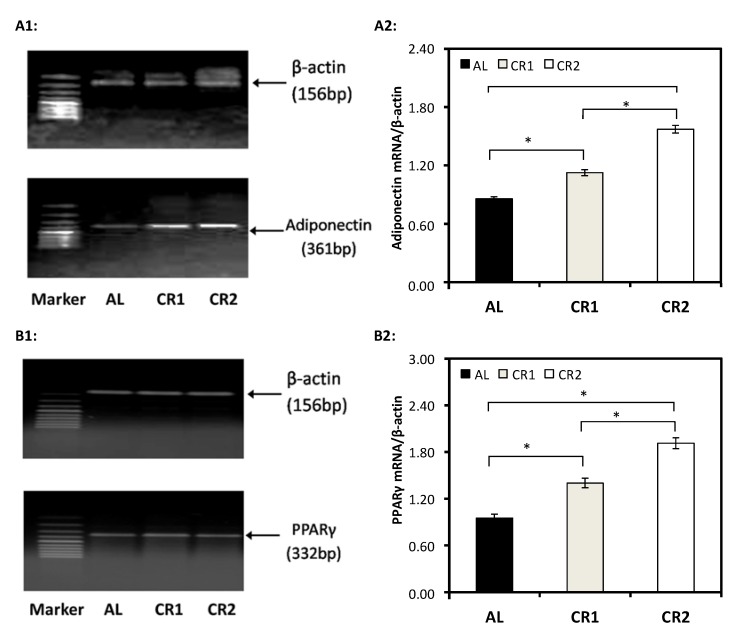
The effect of calorie restriction on the expressions of adiponectin and PPARγ in adipose tissues. (**A1**, **B1**) demonstrate the expression levels of mRNA of β-actin, adiponectin and PPARγ, respectively, quantified by RT-PCR. The expression levels (absorbance) of mRNA of adiponectin and PPARγ were then normalized against those of β-actin (served as loading control) (**A2,** **B2**). One-way ANOVA followed by LSD-test was used to detect significant differences of the means, * *p* < 0.05.

Results obtained from RT-PCR indicated that the expression levels of adiponectin mRNA and PPARγ mRNA in CR groups were significantly higher than those in AL group (Figure 4). Within two CR groups, adiponectin and PPARγ mRNA levels in CR2 group were significantly higher than those in CR1 group (Figure 4(A2,B2)).

### 3.4. Correlation Analysis

Pearson correlation analysis was performed for mRNA levels of adiponectin, PPARγ with different serum parameters (Table 4). Results showed that no correlation was detected between adiponectin, PPARγ mRNA levels, total cholesterol (*p* > 0.05), and fasting glucose (*p* > 0.05). However, adiponectin, and PPARγ mRNA levels were positively associated with HDL-C (*p* < 0.05) and inversely correlated with body weight (*p* < 0.05), triglyceride (*p* < 0.05) and fasting insulin (*p* < 0.05).

**Table 4:** Correlation analysis of adiponectin mRNA, PPARγ ^†^ mRNA with different serum parameters.

Independent Variables	Adiponectin		PPARγ	
	*r*	*p*	*r*	*p*
Body weight	−0.389	0.001	−0.425	0.005
Triglyceride	−0.345	0.042	−0.532	0.032
Total Cholesterol	0.47	0.624	0.673	0.431
HDL-C ^†^	0.376	0.026	0.354	0.032
Glucose	0.530	0.231	0.492	0.485
Insulin	−0.411	0.003	−0.537	0.013

^†^ Abbreviations: PPAR-γ: peroxisome proliferator activator receptor γ; HDL-C: High density lipoprotein cholesterol.

## 4. Discussion and Conclusions

MetS represents a cluster of conditions including glucose intolerance, hypertension, dyslipidemia, and insulin resistance. CR via carbohydrate reduction has elicited a great deal of interest among nutritionists because studies have shown that carbohydrate restriction can improve biological markers that define MetS. In the present study, we conducted a 12-week CR with different levels of carbohydrate reduction in healthy Wistar rats to investigate the changes in MetS-associated biomarkers as well as the expressions of insulin-sensitive adiponectin and its regulator PPARγ.

Body weight and peripheral adipose tissues (Table 3) in CR groups were significantly lower than the AL group and the change of these parameters were inversely associated with the degree of CR. VAT is associated with the development of insulin resistance, glucose intolerance, dyslipidemia and hypertension, whereas subcutaneous adipose tissue is not [21,22,23,24]. The study conducted by Gerbaix *et al.* has validated that removing perirenal and peri-epididymal adipose tissue in rats appears to be more representative of visceral fat mass as dissection of mesenteric and subcutaneous fat is challenging [20]. Hence, in the present study, visceral fat mass was evaluated as the total weight of perirenal and peri-epididymal adipose tissue (Table 3). Our result indicated that CR via carbohydrate reduction lowered visceral fat mass markedly, even when presented as visceral fat mass %, suggesting the potential beneficial effect of CR via carbohydrate reduction on fat distribution and insulin sensitivity in Wistar rats.

Consistent with the results obtained by previous studies involving carbohydrate restriction [25,26], a 68% carbohydrate reduction in CR2 group led to a significant decrease in serum triglyceride and significant increases in LDL-C, HDL-C and total cholesterol as compared to AL group. Our result suggested that in contrast to traditional carbohydrate restriction diets with a low percentage of carbohydrate or even ketogenic diets, our CR diet with 34% carbohydrate reduction was better at improving lipid profiles, as it not only resulted in an increase in HDL-C and a reduction in triglyceride, but also maintained LDL-C at the same level as those fed with control diets, thereby maintaining the total cholesterol at the same level. Decreases in plasma triglyceride by carbohydrate-restricted diet might be the result of downregulation of hepatic de novo lipogenesis [16,26,27]. In addition, carbohydrate restriction might increase muscle lipoprotein Lipase (LPL), thus enhance triglyceride clearance. Increased tissue expression and activity of LPL may partially explain increases in HDL-C in our experimental groups. Increased LPL-mediated catabolism of triglyceride-rich lipoproteins resulted in transfer of unesterified cholesterol, apoprotein and phospholipid to form mature HDL-C [16,26,27].

It is reported that long-term CR leads to an alteration of glucose homeostasis in humans [16] and rats [15,28,29], resulting in decreased glycemia and insulinemia. Nevertheless, our results demonstrated unchanged plasma glucose in CR groups as compared to the AL group. This finding may be owing to the relatively short experimental period (12-week) and the moderate CR in the present study. When the ingested carbohydrates fail to meet the energy needs of the body, the body starts to mobilize glucose from glycogen storage pools for energy supply, and continues to maintain blood glucose level via gluconeogenesis. The low glycogen storage stimulates insulin action in the body. In good agreement with previous studies [15,28,29], fasting insulin level in the present study demonstrated a decreasing trend with CR but only showed marked reduction in CR2 group. HOMA-IR also manifested a decreasing trend and significant reduction in HOMA-IR was observed in both CR1 and CR2 groups. Maintaining relatively low levels of fasting insulin and HOMA-IR is shown to be beneficial for the prevention and treatment of diabetes.

Adiponectin is considered to have antiatherogenic and antidiabetic effects [30]. In accordance with the findings obtained by Zhu *et al.* [31] and Sung *et al.* [14], our results suggest that though serum adiponectin level was only significantly elevated by 60% calorie-restricted diet, gene expression levels of adiponectin and PPARγ in adipose tissues were proportionally elevated with increased degree of energy restriction (Figure 3 and Figure 4)-lower energy intakes led to higher expression levels. A high level of adiponectin predicts good insulin sensitivity and improves lipid and glucose metabolism [32,33]. Activation of PPARγ can induce the synthesis and secretion of adiponectin as a response element of PPARγ was discovered on the promoter of adiponectin gene [10]. CR might activate PPARγ and thus up-regulate the gene expression of adiponectin. Our result indicated that decreased insulin and HOMA-IR achieved by CR might be a result of increased adiponectin. Consistent with previous findings [7,34], our results demonstrated a significant inverse relationship of VAT adiponectin mRNA level and triglyceride and a significant positive relationship of adiponectin mRNA and HDL-C (Table 4). The present study additionally showed a significant association of PPARγ mRNA with triglyceride and HDL-C. Our correlation analysis suggested that elevated gene expression of adiponectin and PPARγ might lead to elevated level of HDL-C and reduced level of serum triglyceride.

Both low-carbohydrate CR diet [16,35] and low-fat, high-carbohydrate diet [36] have been proposed for the prevention of MetS. Despite the fact that a low-fat, high-carbohydrate diet has been advocated for controlling weight [37], a low-carbohydrate CR diet has gained in popularity. Although the low-fat, high carbohydrate diet was better at reducing total cholesterol and LDL-C [26], it is controversial [38], because it raises plasma triglycerides [39] and may adversely affect LDL composition [40,41]. The greater the amount of carbohydrates that are substituted for fat, the greater the increase in triglycerides [42]. Increasing evidence has pointed to the role of triglycerides in atherogenic risk [43]. Consumption of a low-carbohydrate CR diet, however, as shown in our present study and numerous previous studies, resulted in a reduction in triglycerides due to lack of substrates for triglyceride synthesis in liver. In addition, elevated levels of HDL-C were observed in subjects on low-carbohydrate CR diets [16,44], whereas HDL-C declined in subjects on low fat, high-carbohydrate diets [45]. The elevated HDL-C and reduced triglycerides in plasma are the advantages of a carbohydrate restricted diet over a high carbohydrate diet [24].

The long-term low-carbohydrate CR diet, however, is difficult for patients to comply with. Studies have shown the efficacy of a moderate-carbohydrate CR diet in weight management and improvement of serum lipid profiles [46]. In addition, a moderate-carbohydrate CR diet is more acceptable to people for the prevention and treatment of type 2 diabetes, as a strict carbohydrate restricted diet is not required [47,48]. In the present study, for the first time ever, we identified that a moderate-carbohydrate diet (34% carbohydrate reduction) is not only effective in raising gene expressions of adiponectin and PPARγ that potentially lead to better metabolic conditions but is also better in improving lipid profiles than a low-carbohydrate diet in rats. Our results suggest that a moderate-carbohydrate CR diet can be a new dietary intervention strategy for prevention and treatment of MetS.

There are certainly some limitations in this study. First, the investigation period (12 weeks) may be too short to predict long-term effects of CR on expression of adiponectin, PPARγ and serum parameters. Second, RT-PCR is a semiquantitative technique. Although our conclusions were based on the results of the adiponectin, PPARγ expression and other serum parameters, more quantitative techniques such as real-time PCR for mRNA or Western blotting for protein expression of adiponectin should be used in a future study. Finally, whether the conclusion drawn from rats in the present study can be extrapolated to other organisms, such as humans, remains to be determined.

## References

[1] Verdery R.B., Walford R.L. (1998). Changes in plasma lipids and lipoproteins in humans during a 2-year period of dietary restriction in biosphere 2. Arch. Intern. Med..

[2] Walford R.L., Mock D., Verdery R., MacCallum T. (2002). Calorie restriction in biosphere 2: Alterations in physiologic, hematologic, hormonal, and biochemical parameters in humans restricted for a 2-year period. J. Gerontol. A Biol. Sci. Med. Sci..

[3] Bordone L., Guarente L. (2005). Calorie restriction, sirt1 and metabolism: Understanding longevity. Nat. Rev. Mol. Cell Biol..

[4] Takahashi S., Masuda J., Shimagami H., Ohta Y., Kanda T., Saito K., Kato H. (2011). Mild caloric restriction up-regulates the expression of prohibitin: A proteome study. Biochem. Biophys. Res. Commun..

[5] Oana F., Takeda H., Matsuzawa A., Akahane S., Isaji M., Akahane M. (2005). Adiponectin receptor 2 expression in liver and insulin resistance in db/db mice given a beta3-adrenoceptor agonist. Eur. J. Pharmacol..

[6] Bjorndal B., Burri L., Staalesen V., Skorve J., Berge R.K. (2011). Different adipose depots: Their role in the development of metabolic syndrome and mitochondrial response to hypolipidemic agents. J. Obes..

[7] Sadashiv, Tiwari S., Paul B.N., Kumar S., Chandra A., Dhananjai S., Negi M.P. (2013). Adiponectin mrna in adipose tissue and its association with metabolic risk factors in postmenopausal obese women. Hormones (Athens).

[8] Combs T.P., Berg A.H., Rajala M.W., Klebanov S., Iyengar P., Jimenez-Chillaron J.C., Patti M.E., Klein S.L., Weinstein R.S., Scherer P.E. (2003). Sexual differentiation, pregnancy, calorie restriction, and aging affect the adipocyte-specific secretory protein adiponectin. Diabetes.

[9] Vaiopoulos A.G., Marinou K., Christodoulides C., Koutsilieris M. (2012). The role of adiponectin in human vascular physiology. Int. J. Cardiol..

[10] Iwaki M., Matsuda M., Maeda N., Funahashi T., Matsuzawa Y., Makishima M., Shimomura I. (2003). Induction of adiponectin, a fat-derived antidiabetic and antiatherogenic factor, by nuclear receptors. Diabetes.

[11] Combs T.P., Wagner J.A., Berger J., Doebber T., Wang W.J., Zhang B.B., Tanen M., Berg A.H., O’Rahilly S., Savage D.B. (2002). Induction of adipocyte complement-related protein of 30 kilodaltons by ppargamma agonists: A potential mechanism of insulin sensitization. Endocrinology.

[12] Maeda N., Takahashi M., Funahashi T., Kihara S., Nishizawa H., Kishida K., Nagaretani H., Matsuda M., Komuro R., Ouchi N. (2001). Ppargamma ligands increase expression and plasma concentrations of adiponectin, an adipose-derived protein. Diabetes.

[13] Yang W.S., Jeng C.Y., Wu T.J., Tanaka S., Funahashi T., Matsuzawa Y., Wang J.P., Chen C.L., Tai T.Y., Chuang L.M. (2002). Synthetic peroxisome proliferator-activated receptor-gamma agonist, rosiglitazone, increases plasma levels of adiponectin in type 2 diabetic patients. Diabetes Care.

[14] Sung B., Park S., Yu B.P., Chung H.Y. (2004). Modulation of ppar in aging, inflammation, and calorie restriction. J. Gerontol. A Biol. Sci. Med. Sci..

[15] Zhu M., Miura J., Lu L.X., Bernier M., DeCabo R., Lane M.A., Roth G.S., Ingram D.K. (2004). Circulating adiponectin levels increase in rats on caloric restriction: The potential for insulin sensitization. Exp. Gerontol..

[16] Volek J.S., Fernandez M.L., Feinman R.D., Phinney S.D. (2008). Dietary carbohydrate restriction induces a unique metabolic state positively affecting atherogenic dyslipidemia, fatty acid partitioning, and metabolic syndrome. Prog. Lipid Res..

[17] Reeves P.G., Nielsen F.H., Fahey G.C. (1993). AIN-93 purified diets for laboratory rodents: Final report of the american institute of nutrition ad hoc writing committee on the reformulation of the AIN-76A rodent diet. J. Nutr..

[18] Tanno K., Okamura T., Ohsawa M., Onoda T., Itai K., Sakata K., Nakamura M., Ogawa A., Kawamura K., Okayama A. (2010). Comparison of low-density lipoprotein cholesterol concentrations measured by a direct homogeneous assay and by the friedewald formula in a large community population. Clin. Chim. Acta.

[19] Matthews D.R., Hosker J.P., Rudenski A.S., Naylor B.A., Treacher D.F., Turner R.C. (1985). Homeostasis model assessment: Insulin resistance and beta-cell function from fasting plasma glucose and insulin concentrations in man. Diabetologia.

[20] Gerbaix M., Metz L., Ringot E., Courteix D. (2010). Visceral fat mass determination in rodent: Validation of dual-energy X-ray absorptiometry and anthropometric techniques in fat and lean rats. Lipids Health Dis..

[21] Fox C.S., Massaro J.M., Hoffmann U., Pou K.M., Maurovich-Horvat P., Liu C.Y., Vasan R.S., Murabito J.M., Meigs J.B., Cupples L.A. (2007). Abdominal visceral and subcutaneous adipose tissue compartments: Association with metabolic risk factors in the framingham heart study. Circulation.

[22] Freedland E.S. (2004). Role of a critical visceral adipose tissue threshold (CVATT) in metabolic syndrome: Implications for controlling dietary carbohydrates: A review. Nutr. Metab. (Lond.).

[23] Wajchenberg B.L. (2000). Subcutaneous and visceral adipose tissue: Their relation to the metabolic syndrome. Endocr. Rev..

[24] Sasakabe T., Haimoto H., Umegaki H., Wakai K. (2011). Effects of a moderate low-carbohydrate diet on preferential abdominal fat loss and cardiovascular risk factors in patients with type 2 diabetes. Diabetes Metab. Syndr. Obes..

[25] Sharman M.J., Kraemer W.J., Love D.M., Avery N.G., Gomez A.L., Scheett T.P., Volek J.S. (2002). A ketogenic diet favorably affects serum biomarkers for cardiovascular disease in normal-weight men. J. Nutr..

[26] Volek J.S., Sharman M.J., Forsythe C.E. (2005). Modification of lipoproteins by very low-carbohydrate diets. J. Nutr..

[27] Volek J.S., Sharman M.J., Gomez A.L., Scheett T.P., Kraemer W.J. (2003). An isoenergetic very low carbohydrate diet improves serum HDL cholesterol and triacylglycerol concentrations, the total cholesterol to HDL cholesterol ratio and postprandial pipemic responses compared with a low fat diet in normal weight, normolipidemic women. J. Nutr..

[28] Masoro E.J., McCarter R.J., Katz M.S., McMahan C.A. (1992). Dietary restriction alters characteristics of glucose fuel use. J. Gerontol..

[29] Wetter T.J., Gazdag A.C., Dean D.J., Cartee G.D. (1999). Effect of calorie restriction on *in vivo* glucose metabolism by individual tissues in rats. Am. J. Physiol..

[30] Yang X., Zhang Y., Lin J., Pen A., Ying C., Cao W., Mao L. (2012). A lower proportion of dietary saturated/monounsaturated/polyunsaturated fatty acids reduces the expression of adiponectin in rats fed a high-fat diet. Nutr. Res..

[31] Zhu M., Lee G.D., Ding L., Hu J., Qiu G., de Cabo R., Bernier M., Ingram D.K., Zou S. (2007). Adipogenic signaling in rat white adipose tissue: Modulation by aging and calorie restriction. Exp. Gerontol..

[32] Saltiel A.R., Kahn C.R. (2001). Insulin signalling and the regulation of glucose and lipid metabolism. Nature.

[33] Tschritter O., Fritsche A., Thamer C., Haap M., Shirkavand F., Rahe S., Staiger H., Maerker E., Haring H., Stumvoll M. (2003). Plasma adiponectin concentrations predict insulin sensitivity of both glucose and lipid metabolism. Diabetes.

[34] Yang W.S., Chen M.H., Lee W.J., Lee K.C., Chao C.L., Huang K.C., Chen C.L., Tai T.Y., Chuang L.M. (2003). Adiponectin mRNA levels in the abdominal adipose depots of nondiabetic women. Int. J. Obes. Relat. Metab. Disord..

[35] Accurso A., Bernstein R.K., Dahlqvist A., Draznin B., Feinman R.D., Fine E.J., Gleed A., Jacobs D.B., Larson G., Lustig R.H. (2008). Dietary carbohydrate restriction in type 2 diabetes mellitus and metabolic syndrome: Time for a critical appraisal. Nutr. Metab. (Lond.).

[36] Krauss R.M., Deckelbaum R.J., Ernst N., Fisher E., Howard B.V., Knopp R.H., Kotchen T., Lichtenstein A.H., McGill H.C., Pearson T.A. (1996). Dietary guidelines for healthy american adults: A statement for health professionals from the nutrition committee, American heart association. Circulation.

[37] Halton T.L., Willett W.C., Liu S., Manson J.E., Albert C.M., Rexrode K., Hu F.B. (2006). Low-carbohydrate-diet score and the risk of coronary heart disease in women. N. Engl. J. Med..

[38] Schneeman B.O. (2001). Carbohydrate: Friend or foe? Summary of research needs. J. Nutr..

[39] Parks E.J., Hellerstein M.K. (2000). Carbohydrate-induced hypertriacylglycerolemia: Historical perspective and review of biological mechanisms. Am. J. Clin. Nutr..

[40] Dreon D.M., Fernstrom H.A., Miller B., Krauss R.M. (1994). Low-density lipoprotein subclass patterns and lipoprotein response to a reduced-fat diet in men. FASEB J..

[41] Dreon D.M., Fernstrom H.A., Williams P.T., Krauss R.M. (1999). A very low-fat diet is not associated with improved lipoprotein profiles in men with a predominance of large, low-density lipoproteins. Am. J. Clin. Nutr..

[42] Retzlaff B.M., Walden C.E., Dowdy A.A., McCann B.S., Anderson K.V., Knopp R.H. (1995). Changes in plasma triacylglycerol concentrations among free-living hyperlipidemic men adopting different carbohydrate intakes over 2 y: The dietary alternatives study. Am. J. Clin. Nutr..

[43] Yuan G., Al-Shali K.Z., Hegele R.A. (2007). Hypertriglyceridemia: Its etiology, effects and treatment. CMAJ.

[44] Fontana L., Meyer T.E., Klein S., Holloszy J.O. (2004). Long-term calorie restriction is highly effective in reducing the risk for atherosclerosis in humans. Proc. Natl. Acad. Sci. USA.

[45] Turley M.L., Skeaff C.M., Mann J.I., Cox B. (1998). The effect of a low-fat, high-carbohydrate diet on serum high density lipoprotein cholesterol and triglyceride. Eur. J. Clin. Nutr..

[46] Johnston C.S., Tjonn S.L., Swan P.D., White A., Hutchins H., Sears B. (2006). Ketogenic low-carbohydrate diets have no metabolic advantage over nonketogenic low-carbohydrate diets. Am. J. Clin. Nutr..

[47] Daly M.E., Paisey R., Paisey R., Millward B.A., Eccles C., Williams K., Hammersley S., MacLeod K.M., Gale T.J. (2006). Short-term effects of severe dietary carbohydrate-restriction advice in type 2 diabetes—A randomized controlled trial. Diabet. Med..

[48] Haimoto H., Iwata M., Wakai K., Umegaki H. (2008). Long-term effects of a diet loosely restricting carbohydrates on HbA1c levels, BMI and tapering of sulfonylureas in type 2 diabetes: A 2-year follow-up study. Diabetes Res. Clin. Pract..

